# Benchmarking Medical Information Services Beyond the Unsolicited Requests: A phactMI Benchmarking Survey

**DOI:** 10.1007/s43441-025-00787-x

**Published:** 2025-05-12

**Authors:** Michael J. DeLuca, Rena Rai, Kirtida Pandya, Lillian Chavez, Prachee Satpute, Michael Rocco, Parul Shah, Jen Multari, Michael Cuozzo, Evelyn R. Hermes-DeSantis

**Affiliations:** 1 Eversana, Boston, USA; 2 Johnson & Johnson, Somerset, USA; 3 Sandoz, Princeton, USA; 4 Novartis Corporation, East Hanover, USA; 5 Gilead, San Francisco, USA; 6 Centerport, USA; 7 San Ramon, USA; 8 Boehringer Ingelheim, Ingelheim Am Rhein, Germany; 9 Incyte Corporation, Wilmington, USA; 10 PhactMI, 5931 NW 1st Place, Gainesville, FL 32607 USA

**Keywords:** Medical information, MLR, Benchmark, Strategy/planning, Insights

## Abstract

**Objective:**

Medical Information has a strategic role that extends beyond inquiry management. The Pharma Collaboration for Transparent Medical Information (phactMI™) benchmarking survey of 35 US pharmaceutical companies was conducted to describe the current landscape and future opportunities of other services Medical Information could provide.

**Methods:**

In July 2023, an electronic survey containing 57 closed and open-ended questions was distributed to phactMI member companies. The survey questions addressed demographics, medical review, development of materials, training, patient services, insights, and awareness.

**Results:**

Medical Information is a significant contributor to the medical review of promotional healthcare provider materials (51%), patient materials (52%), and non-promotional medical materials (45%). Medical Information ensures the accuracy of medical information, fact checks and validates claim accuracy. Fifty percent of the respondents are responsible for reviewing and/or contributing to Medical Affairs material for Field Medical. Additionally, Medical Information trains both Field Medical and Sales teams on the Medical Information function, and to a lesser extent, disease state information. The majority (75%) of Medical Information Departments offer patient information. The vast majority (85%) produce and identify insights. Medical Directors, Field Medical, and Scientific Communications/ Publications often receive shared insights. Fewer individuals integrate insights with Field Medical and Medical Directors. Since 2018, Medical Information activities have seen a rise in advisory board presentations, insights reporting, publications, competitive intelligence, disease state education, surveillance, pathway submission, and labelling activities. Building awareness is still an important aspect of Medical Information and most focus on the development of their Medical Information website.

**Conclusion:**

The essential roles and activities of Medical Information Departments support products at every stage. Medical Information participates with multiple functions in evaluating medical materials and there is a growing trend of including Medical Information in the development and review of Medical Affairs materials. Medical Information has expanded its participation in pathway submissions, publications, and labeling activities. This benchmark for Medical Information can provide a potential best practice template for activities. For the future, the three areas to prioritize are: increasing the strategic value and KPIs of Medical Information, integrating and overseeing AI technology in the insights process, and improving internal visibility.

**Supplementary Information:**

The online version contains supplementary material available at 10.1007/s43441-025-00787-x.

## Introduction

One critical responsibility of a Medical Information Department in a pharmaceutical company is to respond to unsolicited medical inquiries from healthcare professionals (HCPs), payors, patients, and carers in a relevant, timely, accurate, and scientifically balanced manner [1]. Additionally, the strategic function of Medical Information extends throughout the product life cycle and beyond responding to inquiries [2]. Other activities encompass promotional review, medical congress support, clinical trial recruitment and outreach, patient support programs, field training, product labeling support, creation of managed care dossiers, publication planning, and other undertakings [1–3].

Pharmaceutical Collaboration for Transparent Medical Information (phactMI™) is a non-profit consortium of Medical Information leaders from the pharmaceutical industry dedicated to elevating the practice of Medical Information. In 2018, phactMI conducted a benchmarking survey on the other services provided by Medical Information Departments of 27 biopharmaceutical companies in the US [1]. According to Patel et al., Medical Information Departments play a crucial role in multiple activities that bring considerable business benefits, such as supporting medical congress booths, providing insights and metrics reports, submitting scientific data to compendia, and delivering Medical Information training for Sales and Medical Affairs [1].

Much has changed in the industry since the original benchmark survey. One change is the growth of phactMI from 27 companies in 2018 to 35 companies in 2023. The objective of this benchmarking survey was to assess the current landscape of other services Medical Information provides compared to 2018.

## Materials and Methods

A working group of 16 phactMI volunteers developed and launched an electronic survey in the Alchemer.com survey tool. The survey was distributed to one contact at each of the 35 US bio/pharmaceutical member companies of phactMI in July 2023. This cross-sectional survey had 57 closed and open-ended questions. Categories covered demographics, medical review, developing Medical Affairs materials, training Medical Affairs and Sales personnel, patient services (including information for patients, carers and the public), other services, insights, and awareness. Skip logic design was used in the survey to remove irrelevant questions based on previous responses. Members of the working group piloted the survey prior to distribution. The survey was open for 90 days with periodic reminders being provided. The system automatically stored responses to allow participants to resume the survey at any point. Information was de-identified and analyzed in aggregate.

The survey consisted of seven sections, focusing on the following domains based on the previous calendar year (January–December 2022):

1. Company demographics including the regional coverage of Medical Information, department size in full-time equivalents (FTEs), and number of therapeutic areas and products supported;
2. Data on the medical review and approval of promotional (HCPs and patients) and non-promotional (Medical Affairs) materials. Questions assessed responsibility for medical review, Medical Information’s role in the review, the number of products, items, staff, and time spent involved in the reviews;
3. Development of Medical Affairs materials for Field Medical. Questions assessed the role of Medical Information on the development and review of materials;
4. Development of training materials for Medical Affairs and Sales personnel. Questions assessed the role of Medical Information, time spent, and quantity of different areas of training;
5. Patient services and other services Medical Information provides. Questions assessed Medical Information’s role in each of the services identified. More detailed questions evaluated the channels for patient information, materials used at medical congress booths, literature surveillance, and new services planned;
6. Insights and tools used for insight identification, compiling of insights, sharing of insights, and use of Artificial Intelligence (AI); and
7. Evaluation of how Medical Information is raising awareness of the department and phactMI (supplementary material for full survey).

All survey questions were optional, and companies responded based upon domains and areas specific to their scope of work. In the results, denominators will change based on the number of companies that responded to each question.

Descriptive statistics and Chi-squared were used to analyze the data as appropriate. Missing data was accounted for in sample size reporting for each question.

## Results

### Organizational Demographics

All 35 (100%) of phactMI member companies took part in the survey with each company responding to specific areas based on their involvement. The number of companies that answered the specific question based on their scope of work is represented by the denominator. There was an even distribution of companies by size with 37% (13/35) representing small companies (revenue < $10 Billion), 29% (10/35) midsize companies (revenue $10–30 Billion), and 34% (12/35) large companies (revenue > $30 Billion). Most (51%, 18/35) provide global coverage for Medical Information, with the rest being divided among US only (23%, 8/35), US as part of regional area (17%, 6/35), US plus Global (6%, 2/35), and US only and as part of a regional area (3%, 1/35).

The average number of internal employees was 25 FTEs, whereas external employees accounted for an average of 13 FTEs, both excluding contact center. While the number for internal FTEs was proportionate to the size of the company, external FTEs were highest in large companies (35 FTEs) followed by small companies (9 FTEs) and then midsize companies (5 FTEs). The number of therapeutic areas supported by Medical Information teams ranged from one area to 15 areas. The number of products ranged from 1 to 327, with an average of 35 current/active products, 18 mature/legacy products, and 11 pipeline products.

### Medical Review and Approval of Promotional and Non-promotional Materials

**Figure 1:**
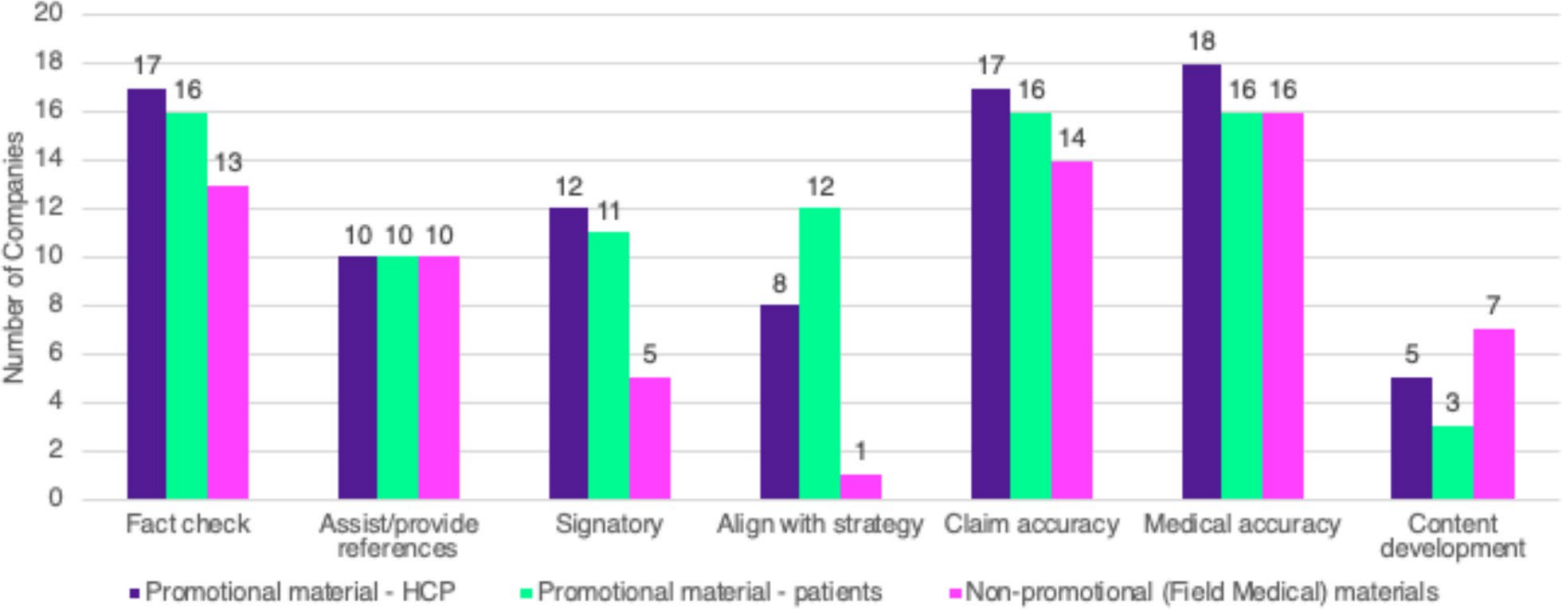
Medical Information’s role in the review process for promotional and non-promotional materials Promotional Materials for HCPs n = 18 Promotional Materials for patients n = 17 Non-promotional (Field Medical) materials n = 16.

While there are a variety of teams involved and responsible for a medical review of promotional HCP and patient materials, and non-promotional (Medical Affairs) materials, Medical Information is a significant contributor. Fifty-one percent (18/35) of participating Medical Information departments are involved in the review of promotional HCP materials), 52% (17/ 33) in promotional patient material, and 45% (14/ 31) in non-promotional material. The primary activities for Medical Information in the review process include medical accuracy (94–100%), claims accuracy (88–94%), and fact checking (81–94%) for promotional HCP or patient and non-promotional materials. (See Fig. 1) Areas where Medical Information teams were less involved were signatory approval (31%) and ensuring strategy alignment (6%) of non-promotional materials. Results also showed a modest decline in the involvement of Medical Information teams in reviewing non-promotional materials, from 56% in 2018 to 45% in 2023. Approximately 70% of respondents stated their companies did not have a benchmark to allocate and quantify staffing needs for medical review of both promotional (73%, 11/15) and non-promotional (69%, 9/13) materials. The majority (53%, 8/15) of companies noted no differences in reviewing promotional HCP or patient materials, and only 40% (6/15) noted that health literacy checks were conducted for promotional patient materials.

**Figure 2:**
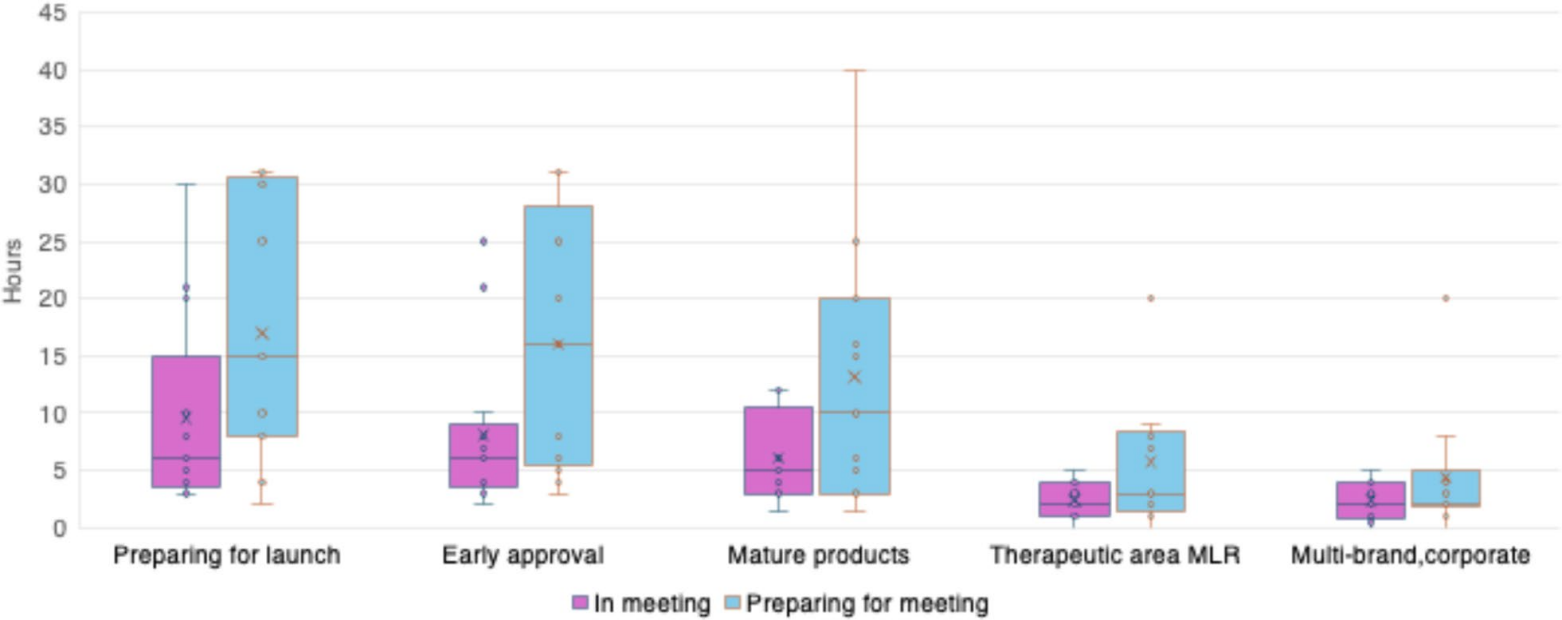
Comparison of time (hours) in meeting to prep time for Medical Legal Regulatory Review committees.

Medical Information teams dedicate substantial time to reviewing promotional materials independently from Medical, Legal, Regulatory (MLR) review meetings. The average time commitment for both preparation and in-meeting review is typically more when preparing for launch and within the first six months post-regulatory product approval. The median number of hours per week is approximately 16 hours reviewing materials prior to meetings and 6 hours in MLR review committee meetings. (See Fig. 2).

### Development of Medical Affairs Materials for Field Medical

**Figure 3:**
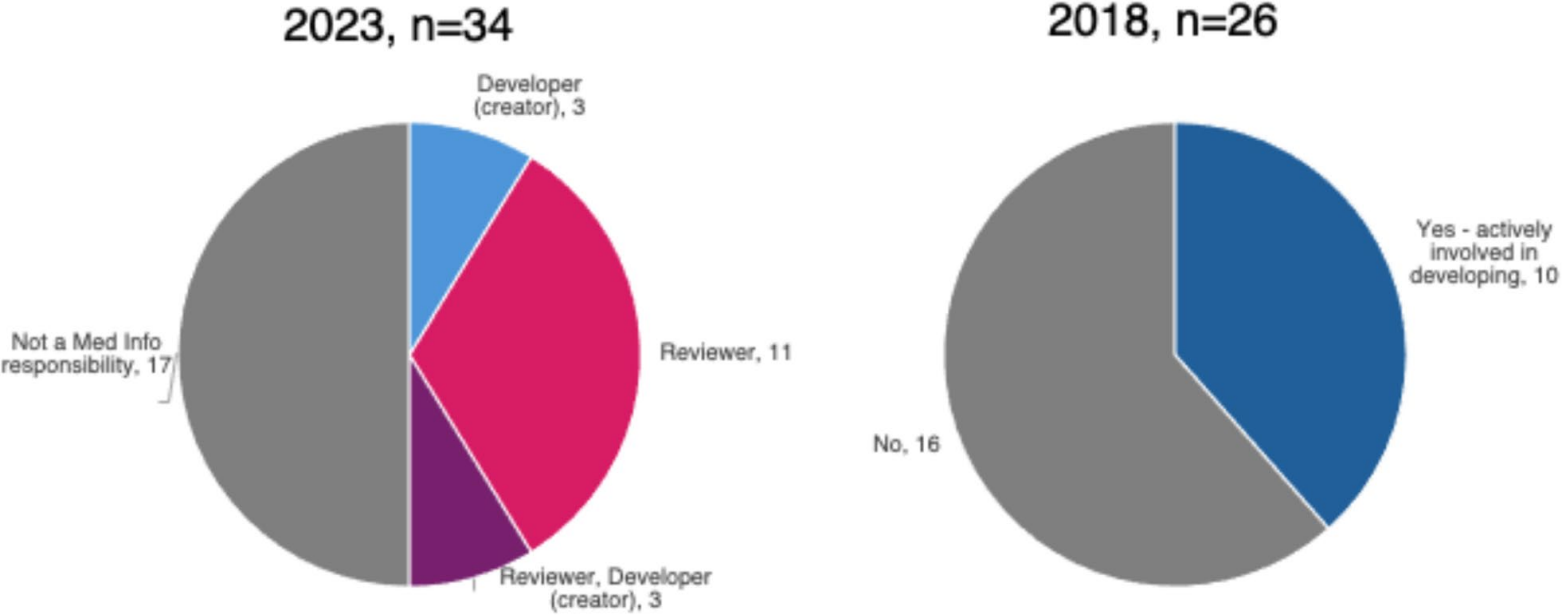
Medical Information involvement in Medical Affairs materials.

In 2023, 50% (17/34) of Medical Information departments reported being involved in developing or reviewing Medical Affairs materials for Field Medical. This was a 12% absolute increase from 2018 when only 38% (10/26) were actively involved with Medical Affairs material. (See Fig. 3) Field Medical materials with Medical Information involvement included infographics (48%, 16/33), slide decks (44%, 15/34), training materials (42%, 13/31), frequently asked questions (FAQs) (42%, 13/31), and videos (34%, 11/32). Where companies are involved, the majority have a reviewer-only role (72–77%), except for infographics where only 38% are reviewer-only. For companies involved in development or development and review (excluding reviewer-only), infographics has the highest involvement (63%, 10/16) followed by slide decks (27%, 4/15) and video (27%, 3/11), and then training materials and FAQs (23%, 3/13 for both).

The Medical Information team spent an average of 60 hours per week in some aspect of the development of the material for an average of 17 brands and 370 items in 2023. Interestingly the number of brands supported has doubled, on average, since 2018 (17 in 2023 vs. 8 in 2018), and the number of items or pieces supported has increased, on average, eightfold (370 in 2023 vs. 47 in 2018).

### Training Materials for Field Medical and Sales

Most (71%, 24/34) companies provided training on the Medical Information function specifically to Field Medical, which was an increase from the 2018 survey of 63% (17/27). Conversely, only 24% (8/34) of the Medical Information departments provided product/disease state training to Field Medical. This was a notable decrease compared to 2018, where 50% (13/26; p = 0.03) reported providing product/disease state training to Field Medical. Similarly, Sales teams also received training on the Medical Information function (74%, 25/34) and product/disease state (21%, 7/34) from Medical Information departments. Additional topics for training by Medical Information Departments included the Medical Information Request Form (MIRF) process, congress coverage, FAQs and compliance topics. Medical Information provided training on these topics to Field Medical (24%, 8/34) and Sales (18%, 6/34).

### Medical Information for Patients

Twenty-five of the 34 companies (74%) responded that they provide medical information to patients. This is a significantly higher number compared with 2018, when only 13 of the 27 companies (48%) responded to these inquiries (p < 0.05).

**Figure 4:**
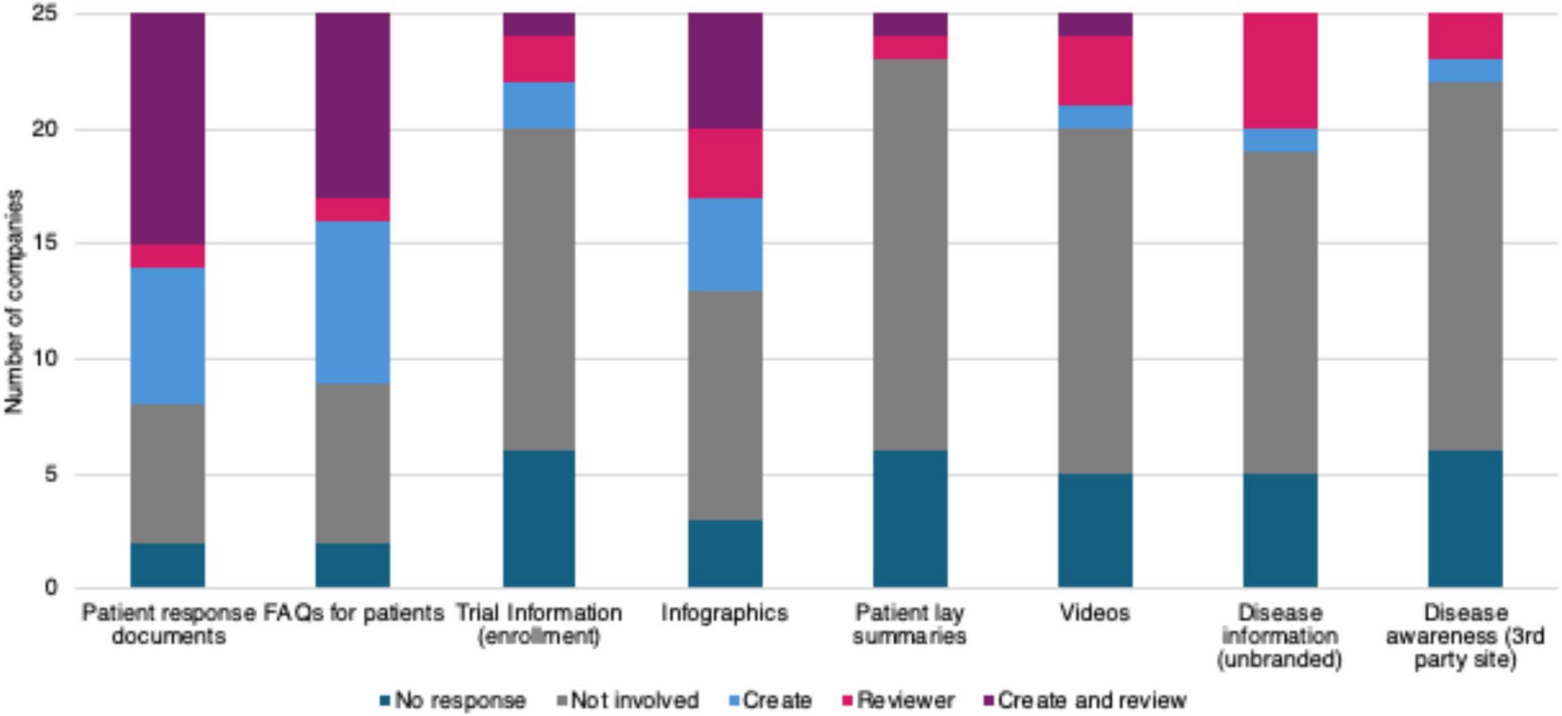
Medical Information staff involvement in patient-centric materials. (n = 25).

Medical Information personnel were primarily involved in the creation and/or review of patient response documents (68%, 17/25), FAQs for patients (64%, 16/25), and infographics (52%, 13/25). Other activities included the creation and/or review of trial information (24%, 6/25), unbranded disease state information (24%, 6/25), videos (20%, 5/25), 3rd party disease awareness information (16%, 4/25), and patient lay summaries (8%, 2/25). (Fig. 4).

**Figure 5:**
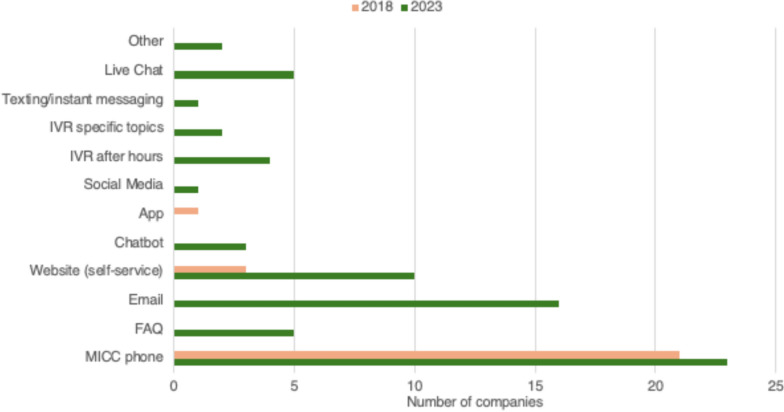
Channels/platforms used by Medical Information to provide information to patients and carers, 2023 vs. 2018. (2023 N = 25; 2018 N = 27).

The majority of companies (92%, 23/25) utilize a Medical Information Contact Center to provide information to patients. Other common communication channels included email (64%, 16/25), self-service websites (40%, 10/25), live chat (20%, 5/25), and FAQs (20%, 5/25). Less common channels included interactive voice response (IVR) for specific topics (16%, 4/25) or after hours (8%, 2/25), chatbots (12%, 3/25), text messaging (4%, 1/25), and social media (4%, 1/25). (See Fig. 5).

When these channel offerings are compared to 2018, the impact of the recent shift to digital engagement is clear. In 2018, no companies cited email, FAQs, chatbots, live chat, text messaging, social media, or IVR as one of their engagement channels. In 2023 there was also an increase in the number of companies that offered Medical Information Contact Center (21 vs 23) and self-service websites (3 vs 10, p < 0.05) for patients.

### Additional Services

Medical Information has customarily offered many services beyond inquiry management. In assessing the current landscape of additional services via this survey, one can appreciate how various areas of services across companies have shifted from 2018 to 2023. Medical Information Departments that are involved with pathway submissions doubled in 2023 (44%, 15/34) compared to 2018 (22%, 6/27), where larger companies have more involvement (73%, 8/11) in pathway submissions compared to midsize (25%, 2/8) and smaller (45%, 5/11) organizations. Similarly, more companies reported involvement in publications in 2023 (38%, 13/34) compared to 2018 (19%, 5/22), as well as labeling activities (2023: 47%, 16/34; 2018: 33%, 9/22). A downward trend was noted for Medical Information team participation in scientific coverage of medical congresses in 2023 (33%, 11/34) compared with 2018 (52%, 14/27). Similarly, there was less involvement in literature search activities in 2023 (79%, 27/34) vs 2018 (93%, 25/27).

Specifically looking at medical congress booth support, 53% (18/34) are collaborators, 21% (7/34) are owners, 21% (7/34) are owners and collaborators, while only 6% (2/34) are not involved in any aspect. The materials displayed in a medical congress booth included interactive tools (80%, 16/20), documents such as publications (55%, 11/20), virtual reality (50%, 10/20), and medical information requests (10%, 2/20). The majority of companies provide information on marketed products (87%, 26/30), pipeline information (87%, 26/30), and clinical trial information (80%, 24/30). Medical Information reviews (41%, 14/ 34), develops (6%, 2/34), or develops and reviews (9%, 3/34) the materials available in a Medical Information booth. When the Medical Information booth is not staffed or there is no Medical personnel at the booth, medical information inquiries are collected via self-service (32%, 11/34), a MIRF (21%, 7/34), QR code to submit the question (6%, 2/34), Field Medical coverage (6%, 2/34), Field Medical business card available (3%, 1/ 34), and webform (3%, 1/ 34); while 27% (9/34) do not collect questions. There was a decrease noted in Medical Information team participation and attendance at sessions of medical congresses from 2018 (52%, 14/27) to 2023 (32%, 11/34).

### Insights

“An insight is the deeper understanding of the why behind trends of information that lead us to determine if an action is warranted” [4]. Insights can lead to strategic initiative by various functions within a pharmaceutical company. Medical Information’s database of customer interactions is a rich resource for insights to inform both department and broader company activities.

A majority (88%, 30/34) of Medical Information Departments generate insights. To appropriately develop insights, multiple sources of data may be necessary. Manual review of questions within the Medical Information inquiry database is most commonly used ( 83%, 25/30) to develop insights. Additionally, respondents also leverage input from front-line agents (60%, 18/30) and Field teams (47%, 14/30). There is clearly an opportunity for companies to consider an automated solution to help generate insights.

**Figure 6:**
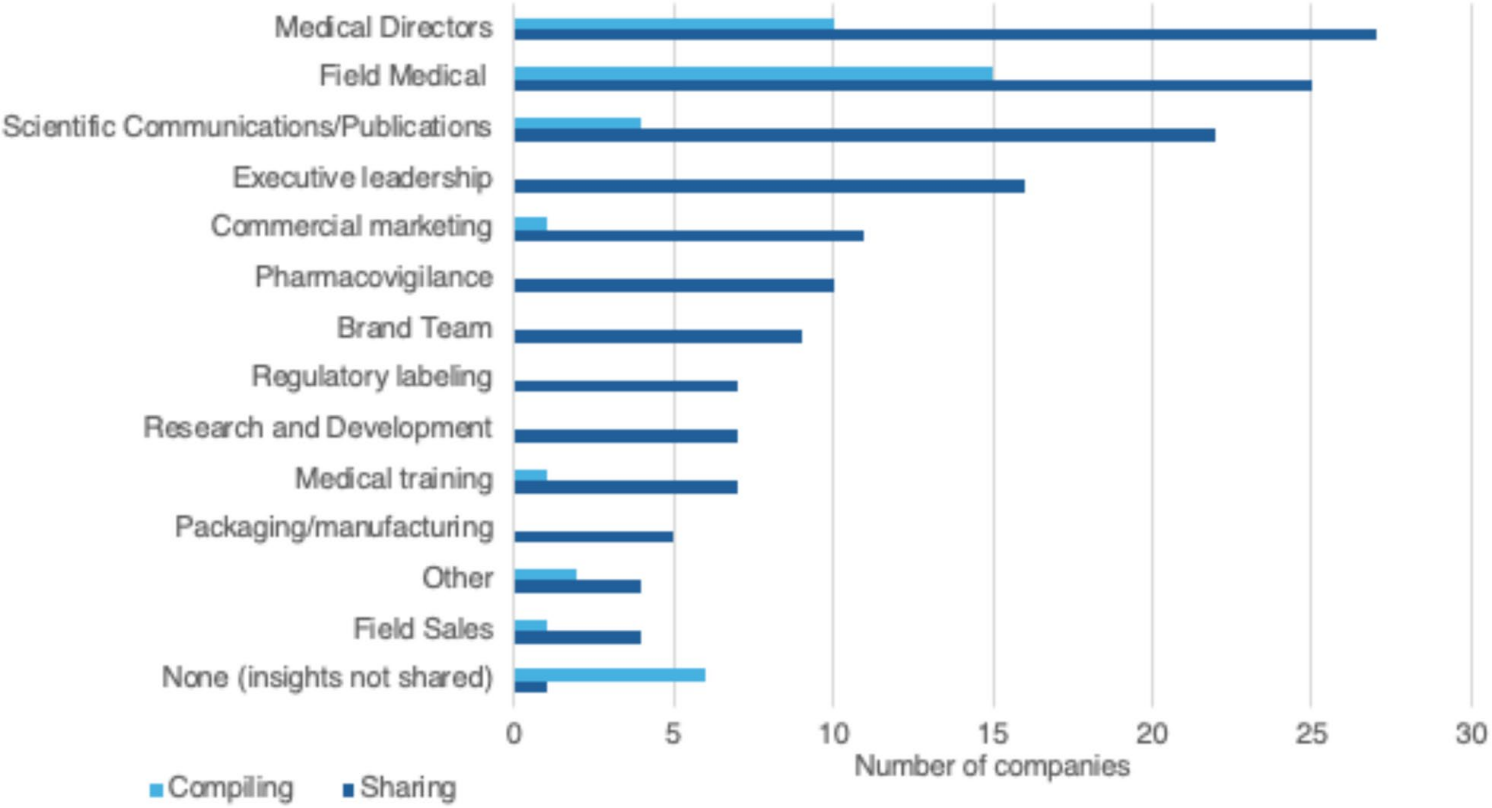
Departments/Functions with which Medical Information shares and/or compiles insights with. Sharing n = 29; Compiling n = 24.

As tools advance, teams are exploring different approaches to collaborate and promptly detect emerging trends. According to the majority of respondents, Medical Information primarily shares data and insights with Medical Directors (93%, 27/29), Field Medical (86%, 25/29), and Scientific Communications/Publications (76%, 22/29). Additionally, they compile insights most frequently with those from Field Medical (63%, 15/24) and Medical Directors (42%, 10/24) compared to other departments. (See Fig. 6) The collaboration of various functions in the pharmaceutical industry is necessary to generate and clarify insights, and using a manual process can help with this. Like other industries, the use of AI in Medical Information is a rising trend. A small number have fully integrated it or are incorporating it into certain parts of their business, while the majority are evaluating or in the process of adopting it.

### Awareness

**Figure 7:**
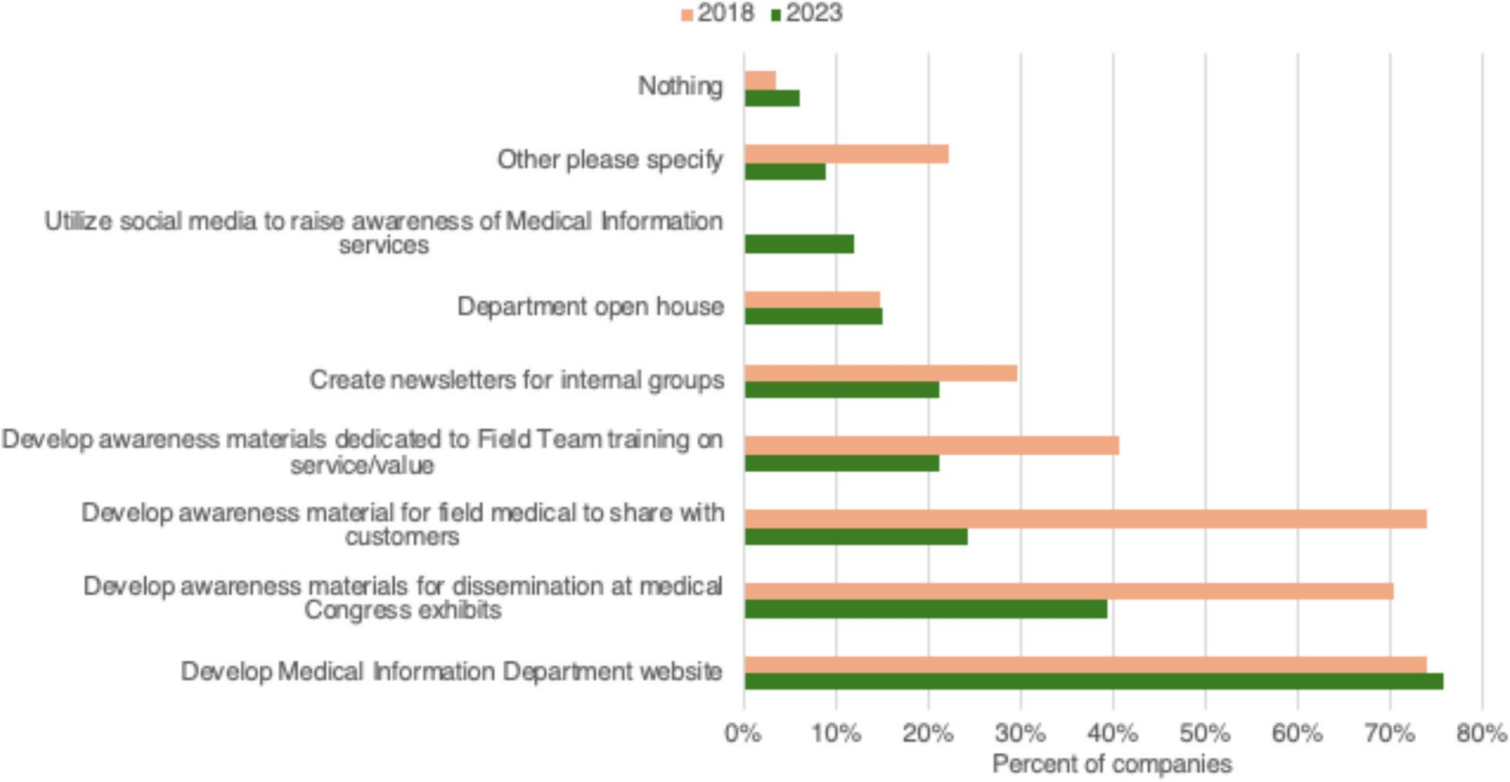
Methods to build awareness concerning Medical Information Services employed by Medical Information Departments in 2023 (n = 33) compared to 2018 (n = 27).

The survey included multiple questions regarding what each member company is doing to raise awareness about their Medical Information services, including if they utilize social media. The most common methods to build awareness were deployment of Medical Information websites (75%, 25/33) and awareness materials for dissemination at medical congress booths (39%, 13/33). Other methods were used less frequently (< 25%) and social media was used by only 12% (4/33) of companies. Most avenues to build awareness were used less frequently than observed in the full benchmark survey phactMI conducted in 2018. [Data on file, phactMI 2018]. (See Fig. 7).

In relation to efforts of member companies to build awareness of phactMI, 39% (13/33) stated that summaries of phactMI meetings and materials were shared with internal teams, followed by 33% (11/33) who provided a link on their Medical Information website and 33% (11/33) who did nothing.

## Discussion

The survey showcases the diverse services offered by Medical Information beyond inquiry management. While certain services have declined since the 2018 benchmarking survey, numerous others have seen growth.

Results indicate that a slightly lower number of Medical Information team members review and approve non-promotional materials compared to promotional materials, which may be attributed to separate review processes and the requirement for a comprehensive Medical, Legal, Regulatory review for medical materials. Additionally, Medical Information could also be the owner and creator of some materials and thus not the reviewer. The growing trend of developing and reviewing Medical Affairs material for Field Medical presents an opportunity for Medical Information specialists to enhance the value of these assets by leveraging their unique customer-centric perspectives and holistic approach to creating medical information responses and materials.

**Figure 8:**
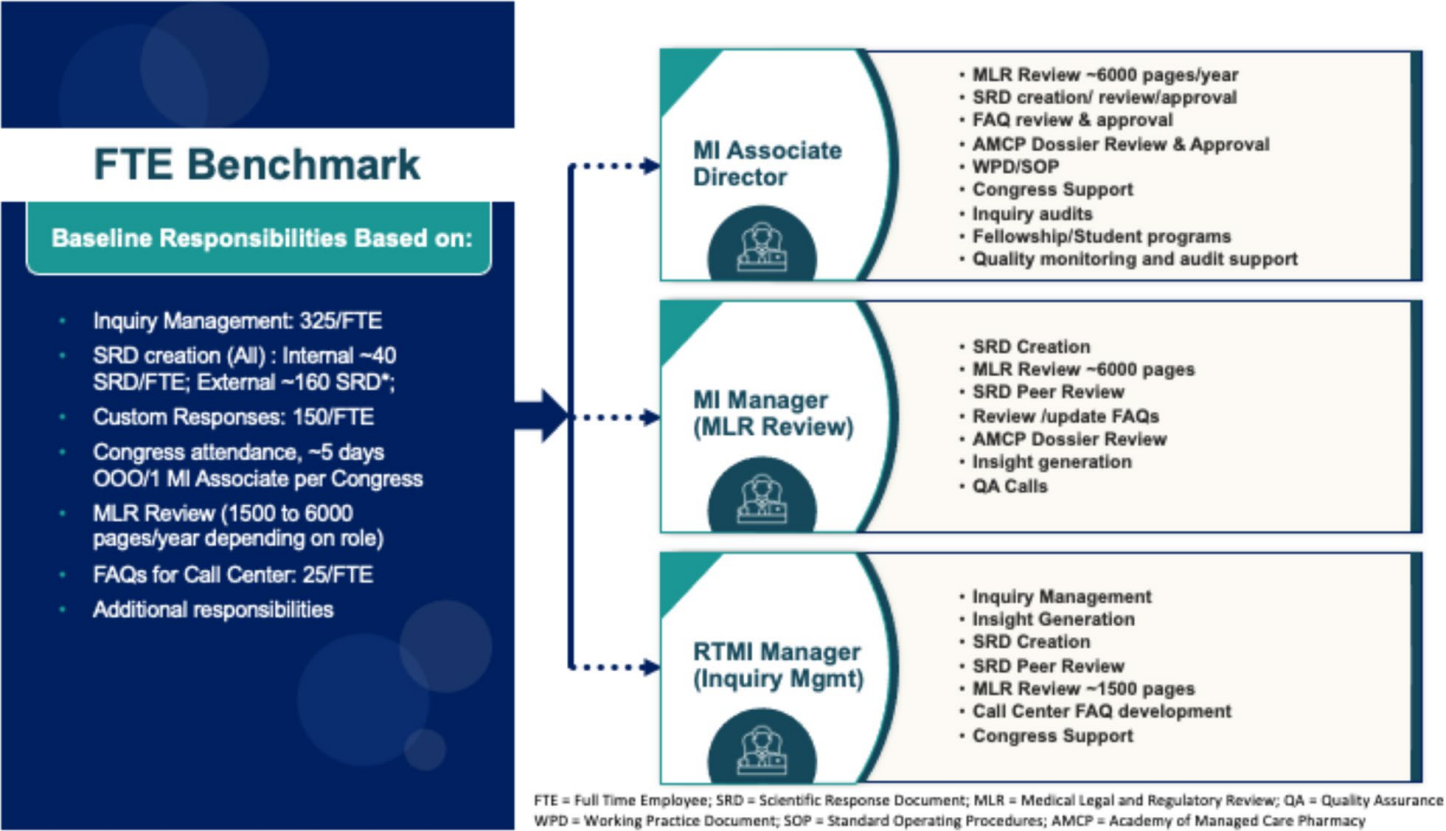
Example benchmarking.

There is a lack of metrics and key performance indicators (KPIs) captured around medical review to ensure adequate funding and resourcing. Companies should develop standards and benchmarks around metrics and KPIs and models to ensure adequate funding and resourcing. However, benchmarks can be subjective based on an organization’s set up, number of levels (Manager, Associate Director, Director, etc.) and may be developed based on several assumptions (see Table 1 and Fig. 8).

**Table 1:** Assumptions for developing benchmarking for resources

Assumptions to consider
• Number of launches annually
• Roles and responsibilities related to the scope of Medical Information vs Medical
• Number of products involving Alliance partnerships
• Core claims compendia
• Scientific communications platforms
• Branded websites
• Visual aid and backgrounders
• Field Medical decks
• Economic models
• Live meetings
• Social media and other digital capabilities including websites, portals, and chatbot functionality
• Congress planning and related activities
• Onboarding/mentoring new employees, Fellows, and Interns
• Other responsibilities

Medical Information’s interaction with patients has grown since 2018. In contrast to 2018, there has been an increase in the number of companies developing self-service web portals for patients (10 vs ~ 3). Medical Information is primarily responsible for patient response documents and FAQ documents related to patient-centric materials, with an increased focus on patient support.

Medical Information actively engages in a wide range of other business pursuits, with some differences being observed based on the size of the company. The number of Medical Information teams that participate in scientific coverage of medical congress decreased from 52% in 2018 to 32% in 2023. It is unfortunate to see this decline, as attendance at these conferences provides an opportunity to meet cross functional colleagues and network with key opinion leaders. However, it may be understandable in an environment of reduction in travel, budget impact, staffing issues, etc.

Uncovering valuable insights can be time consuming, and most companies still rely on manual reviews. Many companies lack a dedicated tool for collecting data from different databases and formulating insights, which can be beneficial to consider. Furthermore, taking a comprehensive approach to insights across the entire organization leads to a deeper understanding of the insight(s). Medical Information currently shares insights with various functions, including Medical Directors, Field Medical, and Scientific Communications/Publications. However, the compilation of insights tends to be limited, mainly focusing on Field Medical. Medical Information can play a crucial role in compiling insights across Medical Affairs and beyond.

Developing a comprehensive and consistent strategy for raising awareness of Medical Information services is another area for companies to prioritize. Since most companies now have a Medical Information website, it is crucial to raise awareness about it and the services it offers. phactMI also has the opportunity to develop recommendations, best practices, and materials to help companies build awareness of Medical Information services and phactMI. Awareness-building techniques decreased in 2023 compared to the outcomes of 2018. Social media use by Medical Information departments was reported to be low; however, this was inconsistent with results from the Medical Information technology benchmarking survey [5]. The focus of these social media pages might be more on Medical Affairs rather than solely on Medical Information, which could account for the difference in results.

## Limitations

The direct comparison to 2018 data may have been limited by factors associated with the specific wording and interpretation of questions by survey respondents. This was evident in several instances where respondents provided unclear or inconsistent responses, making it difficult to draw accurate conclusions. Additionally, the wording of certain questions may have influenced respondents, further complicating the comparison process.

## Conclusion / Suggestions

Medical Information Departments engage in various activities to support products throughout their lifespan, from the research pipeline to maturity. The involvement of multiple functions in evaluating medical materials underscores the significance of Medical Information. There is a growing trend of including Medical Information in the development or review of Medical Affairs materials. While Medical Information trains both Medical Affairs and the Sales team on the Medical Information function, disease state training is not given precedence. There was a notable rise in the involvement in developing materials for patients. Medical Information has expanded its participation in pathway submissions, publications, and labeling activities. Insights is a common focus for companies, with most sharing their findings and many working together to compile insights. Insights is perfectly suited for AI to identify and assess customer sentiment, generate scientific content, manage workflows, and analyze structured and unstructured data from various Medical Affairs functions, providing metrics and trend reporting. Many initiatives to raise awareness about Medical Information may need to be enhanced. By utilizing benchmarking survey results, Medical Information groups can identify ways to enhance their support for customers and business partners.

Based on this benchmarking survey, the authors recommend that Medical Information Departments focus on the following areas:

1. Amplifying strategic value and KPIs of Medical Information by highlighting their unique customer-centric perspectives and leveraging synergies. Medical Information professionals possess inherent abilities for thorough literature research and evaluation, offering broad perspectives and strategic insights.
2. Implementing and managing AI technology in the insights process allows for identification and collaboration across functional areas, leading to improved feedback and cross-functional collaboration.
3. Enhancing the visibility of Medical Information Services within each company through the establishment of best practices such as self-advocacy, task force representation, and aligning with the bigger picture of Medical Affairs.

## Supplementary Information

Below is the link to the electronic supplementary material.

Supplementary file1 (XLSX 35 KB)

Supplementary file2 (DOCX 31 KB)

## Data Availability

Data is provided in the supplementary material.
