# Supplementary material for: Benchmarking Medical Information Services Beyond the Unsolicited Requests: A phactMI Benchmarking Survey
Source: Ther Innov Regul Sci. 2025 May 12;59(4):848–58. doi: 10.1007/s43441-025-00787-x (PMC12181120; doi:10.1007/s43441-025-00787-x)
Supplement: Supplementary file 2 — Supplementary file2 (DOCX 31 KB) [file 43441_2025_787_MOESM2_ESM.docx]

SUPPLEMENTAL MATERIALS

**Benchmark Survey: Other Services Provided by Medical Information**

**Purpose:** to serve as a follow-up to the 2018 benchmark survey^1^ with the objective of assessing the current landscape of other services that Medical Information provides beyond inquiry management. This survey will refer to fiscal year (FY) 2022 activities. The results of this survey will be used to highlight the impact of Medical Information across member organizations.

**Company background**

1. Please provide your company name (for tracking purposes only)
2. Which best describes your Medical Information Department? Select all that apply
   1. US only
   2. US as part of regional area
   3. Global
3. What is the size of your Medical Information Department (excluding call center)?
   1. Internal number of Full-time equivalents (FTEs)
   2. External (vendor) number of FTEs
4. How many therapeutic areas are supported by the Medical Information team?
   1. 1
   2. 2-3
   3. 4-5
   4. 6-7
   5. 8-9
   6. 10 or more please provide number
5. How many products are supported by the Medical Information team?
   1. Current/active products
   2. Mature or legacy products
   3. Pipeline products

**Other services**

**Medical review and approval of promotional and non-promotional material**

1. What group or department is responsible for the following?

|  | medical review of promotional materials – for healthcare professionals (HCPs) | medical review of promotional materials – for patients/consumers | medical review of non-promotional (Field Medical/Medical Affairs) materials |
| --- | --- | --- | --- |
| Medical Information | [go to question 7] | [go to question 7] | [go to question 7] |
| Medical Directors |  |  |  |
| Dedicated Medical Review Team (not including Medical Information) |  |  |  |
| Medical Communications |  |  |  |
| Other (please specify) |  |  |  |

[if Medical Information is not selected, go to question 21]

1. What is the role of Medical Information in the review process?

|  | HCP promotional | Consumer promotional | Non- promotional |
| --- | --- | --- | --- |
| Fact Check |  |  |  |
| Assisting/ provide references |  |  |  |
| Signatory |  |  |  |
| Aligning with strategy |  |  |  |
| Claim accuracy |  |  |  |
| Medical accuracy |  |  |  |
| Content development |  |  |  |
| Other: please specify here: |  |  |  |

**Reviewing medical affairs materials**

1. Does medical information review Field Medical / Medical Affairs (non promotional) material?
   1. Yes
   2. No [go to question 13]
2. For how many total products did the Medical Information Department review Field Medical/Medical Affairs (non-promotional) material in fiscal year FY 2022?
3. How many items/pieces of Field Medical/Medical Affairs (non-promotional) material did the Medical Information Department review during FY 2022?
4. How many of your staff were involved in the review process for Field Medical/Medical Affairs (non-promotional) material?
5. Do you have a benchmark to allocate/quantify staffing for reviewing Field Medical/Medical Affairs (non-promotional) material?
   1. Yes (please explain what assumptions were used to develop this benchmark)
   2. No

**Review of promotional material (both HCP and patient)**

1. Does Medical Information review promotional (either HCP or patient) material?
   1. Yes
   2. No [go to question 21]
2. For how many total products did the Medical Information Department review promotional material for HCPs and/or patients in FY 2022?
3. How many items/pieces of promotional material for HCPs and/or patients did your Medical Information Department review in FY 2022?
4. How many of your staff were involved in the review process for promotional materials (HCP and/or patient)?
5. Do you have a benchmark to allocate/quantify staffing for reviewing promotional materials (HCP and/or patient)?
   1. Yes (please explain what assumptions were used to develop this benchmark)
   2. No

**Any promotional review**

1. Is there a difference in the process of reviewing HCP promotional and patient promotional material?
   1. No difference
   2. Includes health literacy checks for patient promotional material (not HCPs)
   3. Consumer specific experts involved in review of patient promotional material
   4. Other differences (please explain)

1. On average, how many hours are spent (per week) in meetings for promotional review?
   1. Preparing for launch
   2. Early approved products (1^st^ 6 months)
   3. Maintenance/mature products (over 6 months)
   4. Therapeutic area Promotional review committee (PRC) (non-product based)
   5. Multi-brand, corporate level

1. On average, how many hours are spent (per week) in preparation (reviews, pre-reviews, concept review, etc.) for promotional review meetings?
   1. Preparing for launch
   2. Early approved products (1^st^ 6 months)
   3. Maintenance/mature products (over 6 months)
   4. Therapeutic area PRC (non-product based)
   5. Multibrand, corporate level

**Development of medical affairs materials for Field medical**

1. What is Medical Information’s role in Medical Affairs materials for Field Medical? (select all that apply)
   1. Reviewer
   2. Developer (creator)
   3. Not a Medical Information responsibility [go to question 26]
2. How is your Medical Information staff actively involved with Medical Affairs materials for Field Medical? (select all that apply)

| Deliverable | Reviewer | Developer | Not involved |
| --- | --- | --- | --- |
| Slide decks |  |  |  |
| Training materials |  |  |  |
| Frequently asked questions (FAQs) |  |  |  |
| Infographics |  |  |  |
| Videos |  |  |  |
| Other (please specify) |  |  |  |

1. On average, how many hours are spent (per week) on Medical Affairs materials for Field Medical?
2. For how many brands did you update and create Medical Affairs materials for Field Medical during FY 2022?
3. How many items did your Medical Information Department create and update Medical Affairs materials during FY 2022?

**Training: Medical Affairs**

1. Are Medical Information staff involved in training Medical Affairs about the Medical Information function?
   1. Yes
   2. No [go to question 29]
2. On average, how many hours per month (preparing and presenting) are spent on training Medical Affairs about the Medical Information function by Medical Information staff?
3. How many trainings to Medical Affairs concerning the Medical Information function did Medical Information staff deliver during 2022 fiscal year?
4. Are Medical Information staff involved in training Medical Affairs on product(s) and/or disease state(s)?
   1. Yes
   2. No [go to question 32]
5. On average how many hours per month (preparing and presenting) are spent on training Medical Affairs on product(s) and/or disease state(s) by Medical Information staff?
6. How many trainings to Medical Affairs on product(s) and/or disease state(s) did Medical Information staff deliver during FY 2022?
7. Are Medical Information staff involved in training Medical Affairs on other topics?
   1. Yes, please explain
   2. No

**Training- sales force**

1. Are Medical Information staff involved in training the Sales Force about the Medical Information function?
   1. Yes
   2. No [go to question 36]
2. On average how many hours per month (preparing and presenting) are spent training the Sales Force about the Medical Information function by Medical Information staff?
3. How many trainings to the Sales Force concerning the Medical Information function did Medical Information staff deliver during FY 2022?
4. Are Medical Information staff involved in training the Sales Force on product(s) and/or disease state(s)?
   1. Yes
   2. No [go to question 39]
5. On average how many hours per month (preparing and presenting) are spent training the Sales Force on product(s) and/or disease state(s) by Medical Information staff?
6. How many trainings to the Sales Force on product(s) and/or disease state(s) did Medical Information staff deliver during FY 2022?
7. Are Medical Information staff involved in training the Sales Force on other topics?
   1. Yes, please explain
   2. No

**Services for consumers**

1. Does the Medical Information Department provide medical information to consumers (patients/caregivers/general public)?
   1. Yes
   2. No [go to question 43]
2. Which channels/platforms are used by Medical Information to provide information to consumers? (select all that apply)
   1. Website (self-service)
   2. Chatbot
   3. App
   4. Social Media
   5. Phone – Medical Information Contact center (for response documents.
   6. Frequently asked questions (FAQ)
   7. Interactive voice response (IVR) for after-hours service
   8. IVR for specific topics
   9. Email
   10. Texting/instant messaging
   11. live chat
   12. Other (please explain)

1. Is your Medical Information staff involved in consumer-centric materials? (select all that apply)

|  | Response document | FAQ | Trial information (for trial enrollment) | Infographic | Patient lay summaries (publications and posters) | Videos | Disease information (unbranded site) | Disease awareness, 3^rd^ party site (unbranded site) | other |
| --- | --- | --- | --- | --- | --- | --- | --- | --- | --- |
| Create |  |  |  |  |  |  |  |  |  |
| Review |  |  |  |  |  |  |  |  |  |
| Not involved |  |  |  |  |  |  |  |  |  |

**Additional services offered**

1. Are Medical Information staff involved with any of the following activities? Select all that apply

| **Activity** | **Owner** | **Collaborate** | **Not Involved** |
| --- | --- | --- | --- |
| Congress booth support |  |  |  |
| Present/support advisory boards |  |  |  |
| Insight metrics reporting |  |  |  |
| Publications |  |  |  |
| Competitive intelligence |  |  |  |
| Disease state education |  |  |  |
| Literature surveillance activity |  |  |  |
| MI newsletter for internal staff |  |  |  |
| Compendium submission |  |  |  |
| Pathway submission |  |  |  |
| AMCP dossier/other payer information (1-page summaries; testimonials) |  |  |  |
| Labeling activities |  |  |  |
| Response to OSHU/DERP reports |  |  |  |
| ICER submission and reporting |  |  |  |
| Other, please specify: |  |  |  |

Abbreviations: AMCP = Academy of Managed Care Pharmacy; ICER = Institute for Clinical and Economic Review; OSHU/DERP = Oregon Health & Science University/The Drug Effectiveness Review Project.

1. If the Department owns or collaborates on Congress booth support, what materials are displayed in the Medical Information booth and is the interaction with Congress attendees documented?

| **Medical Booth Material** | **Displayed** | **Interaction Documented** |
| --- | --- | --- |
| Video |  |  |
| Display panels |  |  |
| Interactive tool |  |  |
| Documents (e.g., publication) |  |  |
| Virtual reality (e.g., patient journey, mechanism of action) |  |  |
| Other* |  |  |

Other, please specify here:

1. Information provided in a congress booth covers which of the following (select all that apply)
   1. Pipeline information
   2. Marketed products
   3. Clinical trial information
   4. Other

1. Is the Medical Information staff responsible for the materials used in the medical booth?
   1. Developing the materials
   2. Reviewing the materials
   3. Not a Medical Information responsibility
2. Is there a way for Medical Information to collect questions for a congress when there is no Medical person at the booth or when the Medical Information booth is not staffed?
   1. Yes, self-serve
   2. Yes, other please explain
   3. No
3. Does your Medical Information team participate in the scientific coverage (attending sessions) of medical congresses for the company?
   1. Yes
   2. No

For the following questions, the definition of an insight is “the deeper understanding of the why behind trends of information that lead us to determine if an action is warranted.”^2^

1. Does Medical Information generate/identify insights within your organization?
   1. Yes
   2. No [go to question 53]
2. How are insights identified? Select all that apply
   1. Technology – please list products used
   2. Manual review of question details
   3. Input from front line agents
   4. Input from field teams via CRM (Customer Relationship Management)
   5. Other
3. Do you share insights with the following? Select all that apply
   1. None (insights are not shared with any other group)
   2. Scientific Communications/Publications
   3. Field Medical
   4. Medical Director
   5. Commercial – marketing
   6. Field sales
   7. Brand team
   8. Research and Development
   9. Pharmacovigilance
   10. Packaging/manufacturing
   11. Executive Leadership
   12. Medical training
   13. Regulatory/labeling
   14. Other please specify
4. Do you compile insights with the following? Select all that apply
   1. None (insights are not compiled with any other group)
   2. Scientific Communications/Publications
   3. Field Medical
   4. Medical Director
   5. Commercial – marketing
   6. Field sales
   7. Research and Development
   8. Pharmacovigilance
   9. Packaging/manufacturing
   10. Medical training
   11. Regulatory/labeling
   12. Other please specify
5. Where are you in the journey of using artificial intelligence (AI) in insights?
   1. Not using
   2. Considering the use
   3. In the process of assessing the use
   4. In the process of implementation
   5. Using it for some aspects
   6. Fully adopted AI
6. Is your Medical Information staff involved with literature searching?
   1. Yes
   2. No, please list who does the literature searches for you
7. Are you planning to add additional services to your Medical Information Department in the next 1-3 years?
   1. Open text field

1. What is your company doing to raise awareness about its Medical Information services? (select all that apply)
   1. Nothing
   2. Develop awareness material for field medical to share with customers
   3. Develop Medical Information Department website
   4. Develop awareness materials for dissemination at medical Congress exhibits
   5. Utilize social media to raise awareness of Medical Information services
   6. Develop awareness materials dedicated to Field Team training on service/value
   7. Create newsletters for internal groups
   8. Department open house
   9. Other please specify
2. What is your company doing to raise awareness about phactMI?
   1. Nothing
   2. Share summary of meeting/information with internal teams
   3. Link to phactMI website from Medical Information Department website
   4. Other please specify

Thank you for your time and participation in this survey. Your input is very important.

^1^ Patel M, Jindia L, Fung S, Kadowski R, Marasigan K. pharma collaboration for transparent medical information (phactMI) benchmark study: trends, drivers, and value of product support activities, key performance indicators, and other medical Information Services: insights from a survey of 27 US pharmaceutical medical information departments. Ther Innov Regul Sci 2020; 54: 1275- 1281

^2^ Cadogan AA, Lau J, Wnorowski S, Kelsch GR, Oreper J, Chevez L, Weidman JJ, Hermes-DeSantis ER. Defining insights. Ther Innov Regul Sci*.* 2023;57:1229-1237. <https://doi.org/10.1007/s43441-023-00554-w>
